# Le neurofibrome pré sacré solitaire géant: cause rare de masse pelvienne chez la femme

**DOI:** 10.11604/pamj.2014.17.288.3312

**Published:** 2014-04-15

**Authors:** Majdouline Boujoual, Hafid Hachi, Mohammed Alami Merrouni, Basma El khannoussi, Abdeslam Bougtab

**Affiliations:** 1 Gynécologie Obstétrique, Faculté de Médecine et de Pharmacie d’‘Oujda, Université de Oujda, Oujda, Maroc; 2 Chirurgie, Institut National d'Oncologie Rabat, Maroc; 3 Centre de Radiologie Ibn Sina, Tanger, Maroc; 4 Anatomie pathologique, Institut National d'Oncologie, Rabat, Maroc

**Keywords:** Masse pelvienne, Neurofibrome, Imagerie, Anatomie Pathologique, Chirurgie, pelvic mass, Neurofibroma, imagery, Pathology, surgery

## Abstract

Le neurofibrome présacré solitaire est une tumeur ectodermiquetrès rare tant par sa fréquence que par sa localisation, souvent pauci-symptomatiquejusqu’à l'atteinte de taille importante, d'accès difficile et de diagnostic erroné. L'imagerie préopératoirejoue un rôle essentieldans la prise en charge. Son diagnostic est immuno-histologique. Son traitement chirurgical est basé sur l'exérèse complète à marges saines. Nous rapportons l'observation d'une patiente de 46 ans, ayant été opérée pour suspicion de fibrome utérin sous séreux, dont l'exploration chirurgicale a confirméune tumeur rétropéritonéale et présacrée. L'IRM post opératoire a précisé ses rapports anatomiques. La reprise chirurgicalea permis l'exérèse complète de la tumeur sans lésions des organes adjacents. L'histologie et l’étude immuno-histochimique ont conclu à un neurofibrome.

## Introduction

Les Neurofibromes sont des tumeurs neurogènes non encapsulées issues des gaines nerveuses représentant 5% des tumeurs bénignes des tissus mous [1]. Ils siègent le plus souvent au niveau de la région thoracique suivie de la région cervicale et lombaire, 1 à 5% des cas seulement se localisent au niveau de la région pré sacrée [2]. Ils sont pauci ou asymptomatiques, d'accès difficile et de diagnostic le plus souvent erroné ou fait à un stade relativement avancé [2, 3]. L′imagerie préopératoire joue un rôle essentiel dans la planification du traitement chirurgical [4]. Sa prise en charge doit être multidisciplinaire dans une structure hospitalière tertiaire en raison de la complexité anatomique de cette région [3, 4]. Nous rapportons un nouveau cas de neurofibrome présacré solitaire géant présentant le tableau de masse pelvienne. Son extrême rareté, ses difficultés diagnostiques, thérapeutiques et évolutives nous ontincitésà rapporter cette observation.

## Patient et observation

**Figure 1:**
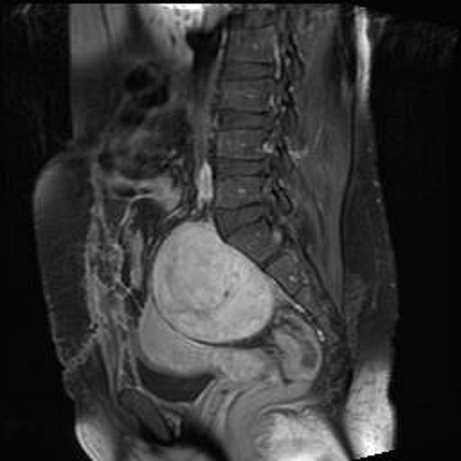
IRM en coupe sagittale montrant une masse rétro utérine et pré sacrée de 91 x 86 x 78 mm, d'intensité légèrement supérieure à celle du muscle en pondération T1

**Figure 2:**
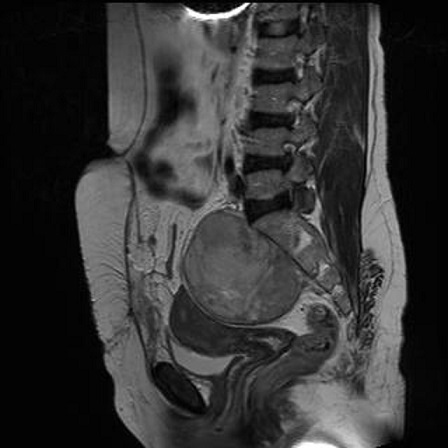
IRM en coupe sagittale montrant un aspect hyper intense de la masse en pondération T2 avec des plages hypo intenses centrales

Patiente âgée de 46 ans, 4ème geste 4ème pare, présentant depuis une année des douleurs pelviennes avec sensation de pesanteur associées à une constipation et pollakiurie. Son exploration échographique initiale était en faveur d'un fibrome utérin sous séreux postérieur. La patiente a été alors opérée, son exploration chirurgicale a découvert une tumeur rétro péritonéale et pré sacrée indépendante des organes génitaux et du tube digestif, faisant 10cm de grand axe. La biopsie n'a pas été réalisée du fait du risque hémorragique. L'IRM post opératoire a mis en évidence en rétro utérin et en avant du sacrum une masse de 91x86x78 mm, d'intensité légèrement supérieure à celle du muscle en pondération T1 et hyper intense en T2 avec des plages hypo intenses centrales et un rehaussement intense et hétérogène après injection de Gadolinium. Par ailleurs, l'utérus était refoulé en avant sans signe d'envahissement. En arrière, la tumeur était accolée au sacrum, sans modification de la structure osseuse (Figure 1, Figure 2).

**Figure 3:**
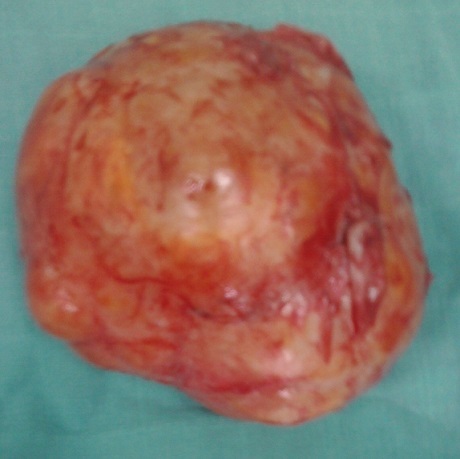
Aspect macroscopique de la pièce opératoire

**Figure 4:**
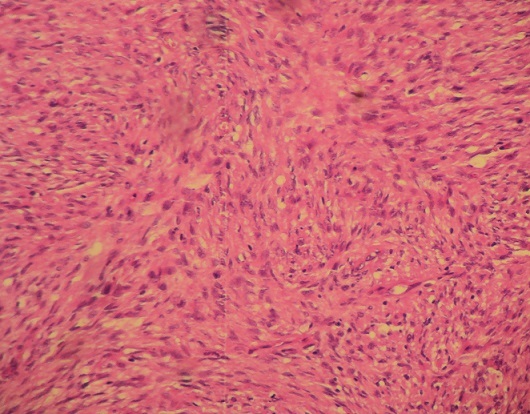
Neurofibrome pré sacré: prolifération fusocellulaire HEX10

**Figure 5:**
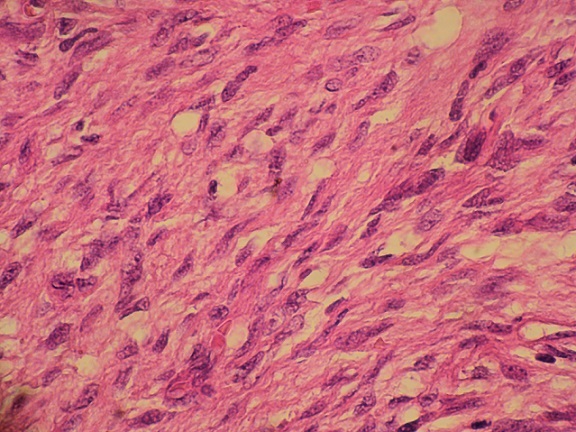
Neurofibrome pré sacré: aspect fusocellulaire avec faisceaux entrecroisés de cellules fusiformes aux noyaux sombres et ondulés, mêlés à des bandes de collagène HEX40

La patiente nous a été référée pour reprise chirurgicale. Son examen neurologique et cutané était normal. Les touchers pelviens ont objectivé une masse rétro utérine de consistance ferme, peu mobilisable, avec sillon de séparation de l'utérus. Une laparotomie exploratrice a été réalisée par une incision médiane, retrouvant une tumeur de l'espace retro péritonéal et pré sacré, ferme, de couleur rosée et ayant un plan de clivage par rapport aux organes adjacents ce qui a permis son exérèse complète. La tumeur pesait 253g et mesurait 10x8 cm de diamètre (Figure 3). Son examen histologique a montré une prolifération fusocellulaire, faite de faisceaux entrecroisés de cellules fusiformes aux noyaux sombres et ondulés, mêlés à des bandes de collagène, sans activité mitotique. L'anticorps anti-PS 100 a montré un marquage diffus au niveau des cellules tumorales évoquant le diagnostic de neurofibrome (Figure 4, Figure 5).

Les suites opératoires étaient simples. Aucun traitement complémentaire n′a été envisagé. Sa surveillance post opératoire n'a pas montré de récidive locale.

## Discussion

Les masses pelviennes rétro péritonéales peuvent être confondues avec les masses d'origine gynécologique lors de l'examen clinique et de l'imagerie préopératoire [1]. Il peut s'agir de tumeurs neurales (schwannome, neurofibrome), graisseuses (lipome, liposarcome),desmoïdes, ganglionnaires (lymphomes) ou vasculaires (hemangiopéricytome, angiosarcome) [5]. Le neurofibrome est une tumeur ectodermique rare qui touche exceptionnellement le rétro péritoine. Il peut être solitaire, c'est le cas de notre patiente, ou souvent multiple associé à la neurofibromatose type 1 (NF1) [6, 7]. Cette tumeur à croissance lente, peut s’étendre dans le rétropéritoine, l'espace pré sacré et les tissus mous, sans dégénérer à l'exception de 4 à 11% des cas associés à une NF1[2]. Notre cas est rare, du fait de la rareté de la localisation pré sacrée chez les patients sansNF1 [8].

La plupart des cas se manifestent de la 2^ème^ à la 4^ème^ décade, alors que ceux associés à la NF1 surviennent àun âge plus précoce [1]. Ces tumeurs demeurent souvent pauci-symptomatiques jusqu′à l'atteinte de taille extrêmement importante[9], entrainant alors des douleurs à type de pesanteur, des troubles digestifs ou urinaires à type d′hématurie, pollakiurie, dysurie voire colique néphrétique. Certains cas peuvent même se compliquer d'un hématome rétropéritonéal massif.

En échographie, le neurofibrome apparaît comme une masse bien limitée, hypo échogène homogène avec renforcement postérieur [1, 6]. La TDM permet d’étudier son extension tumorale notamment au plexus sacré. Toutefois, elle ne permet souvent pas de différencier le neurofibrome géant du neurofibrosarcome. Quant à l'IRM, elle précise le siège exact de la tumeur ainsi que ses rapports aux organes de voisinage. En pondération T1, son intensité est légèrement supérieure à celle du muscle; alors qu'elle est hyper intense en T2. Après injection du gadolinium, sa prise est hétérogène avec légère hypo intensité centrale en T1 [2]. Dans notre cas, le diagnostic de fibrome utérin sous séreux postérieur suspecté en pré opératoire, a été redressé lors de la laparotomie et de l'IRM post opératoire qui était caractéristique du neurofibrome.

La ponction-biopsie transpariétale écho ou scanoguidée est proscrite par certains auteurs du fait du risque d'erreurs diagnostic, d'hémorragie, d'infection et de dissémination tumorale. Elle devrait être réservée aux lésionsnon résécablesnotamment pour entreprendre un traitement adjuvant [4, 6, 10].

Sur le plan anatomopathologique, le neurofibrome est composé de cellules polymorphes comprenant les cellules de Schwann, cellules périneurales et fibroblastes qui sont contenues dans une matrice de mucopolysaccharide. Il s′infiltre entre les fascicules du nerf, le long de son trajet, ce qui pourrait rendre sa résection difficile et dangereuse. Sur le plan immunohistochimique, les cellules du neurofibrome réagissent faiblement avec la protéine S-100[6, 10]. Ces données permettent de faire le diagnostic différentiel avec le schwannome dont la prolifération, faite exclusivement de cellules de Schwann, se fait dans l′endonèvre d′un fascicule nerveux avec un immunomarquage de la protéine S-100 positif [6].

La prise en charge doit être multidisciplinaire avec collaboration entre neurochirurgiens, orthopédistes et chirurgiens visceralistes[10, 11]. Le choix de la voie d'abord dépend du degré de développement intra pelvien et intrasacré. En effet, l'abord est antérieur en cas de tumeur à forte composante pré sacrée: c'est le cas de notre patiente. Il est postérieur en cas de composante intra sacrée ou intra durale. Parfois, l'abord peut être combiné par voie abdomino-sacrée [10]. L′exérèse complète avec marges négatives est le traitement de référence [6], elle doit être carcinologique et conservatrice des structures vasculaires, nerveuses, viscérales et osseuses[12], pour éviter des complications chirurgicales sévères, notamment l'hémorragie du plexus veineux pré sacré, les lésions du rectum et les lésions des nerfs sacrés [3]. Toutefois, en cas de tumeur adhérente ou envahissante, l′exérèse pourrait être élargie aux organes adjacents au prix de déficits neurosensoriels [6]. La radiothérapie est généralement proscrite dans les tumeurs bénignes du fait de son risque potentiellement oncogène [10].

## Conclusion

Le neurofibrome pré sacré solitaire est rare, souvent cliniquement muet et peut atteindre des tailles importantes. L'imageriepermet d’évoquer le diagnostic, de délimiter l'extension et de planifier le traitement. L'exérèse chirurgicale complèteà marges saines constitue le traitement de choix.
